# Investigating primary preservice teachers’ ultraviolet radiation awareness and perceived ability to teach sun safety

**DOI:** 10.1002/hpja.401

**Published:** 2020-08-27

**Authors:** Joseph J. Scott, Robyn Johnston, Sally Blane, Mark Strickland, Jill Darby, Elin Gray

**Affiliations:** ^1^ School of Education Edith Cowan University Joondalup WA Australia; ^2^ Cancer Council Western Australia Subaico WA Australia; ^3^ School of Medical and Health Sciences Edith Cowan University Joondalup WA Australia; ^4^ Melanoma Research Group School of Medical and Health Sciences Edith Cowan University Joondalup WA Australia

**Keywords:** behaviours, perceptions, primary, sun safety, teacher education, ultraviolet radiation

## Abstract

**Issue addressed:**

Sun protection practices in Australian primary schools remain inconsistent. Therefore, this study investigates primary PSTs sun protective sun behaviours, ultraviolet (UV) radiation awareness and perceived ability to teach sun safety.

**Methods:**

A convenience sample of undergraduate PSTs (N = 275; mean age = 23.13 years) enrolled at one Western Australian university completed an online survey. Descriptive analyses provided features of the data. Factors associated with sun protection behaviours and perceived knowledge and skill to teach sun safety were explored using multivariable logistic regression models.

**Results:**

Lesser than 10% of participants reported using sun protective measures daily (midday shade use: 6.5%; sunscreen: 7.6%; hat: 4.4%). Only 56.3% reported they understand the UV index, with 68.0% rarely/never using it to aid sun protection. Under half the participants reported they felt they had the knowledge (38.5%) or skills (40%) to effectively teach sun safety in primary schools. Regression analysis revealed gender, undergraduate, year and skin sensitivity were not predictors of UV index use (*P* > .05) or perceived knowledge of sun safety (*P* > .05). Skin sensitivity was the strongest predictor for shade usage (*P* = .02), hat usage (*P* = .05) and perceived skill to teach sun safety (*P* = .02).

**Conclusions:**

Survey data indicate UV radiation is inconsistently understood by PSTs. Many felt that they did not have the required knowledge or skill to teach sun safety effectively.

**So what?:**

Improving PSTs UV radiation knowledge while at university is a potential opportunity to improve sun safety delivery in primary schools. A targeted intervention for PSTs is warranted.

## 1 INTRODUCTION

In 2018, global research concluded that Australia has the highest incident rate of melanoma in the world.^1^ In 2015 alone, 2162 Australians died as a result of skin cancer with melanoma accounting for 1520 of these cases (642 were non‐melanoma related).^2^, ^3^ Evidence suggests that 95% of melanomas are caused by over exposure to ultraviolet (UV) radiation.^4^ Level of UV radiation exposure, the UV index and the effect it has on sunburn is still poorly understood across the Australian population with many people believing that they are at low risk during days where it is overcast or of cooler temperature.^5^, ^6^ Ability to understand the UV index and how to implement effective sun protection strategies remains paramount in reducing harmful UV exposure and risks of developing skin cancer.^6^

Despite widespread sun safety education across Australia, UV radiation awareness and sun protection practices in schools remain inconsistent.^7^, ^8^, ^9^ Primary schools remain a particularly important location for sun safety education programs as it is believed that educating children on effective sun protective behaviours and the dangers of UV radiation could significantly impact behaviour and lower individuals future risk of developing skin cancer.^10^ In addition, school administrators also can implement policies that promote sun protective behaviours for both teaching staff and students while on school grounds.^11^ By encouraging adherence to school sun protection policies, primary school teachers play an important and influential role in childrens’ sun safety health.^12^, ^13^

Educating preservice teachers (PSTs) while at university before they enter schools could be a potential opportunity to improve schools’ approaches to sun protection across Australia by facilitating classroom teaching on the UV Index and modelling positive sun safe behaviours to students.^14^, ^15^ Anecdotal evidence from one previous study (n = 30) suggests that secondary^1^ PST’s knowledge about the UV Index is low.^16^ However, primary^2^ PST’s current level of understanding of UV radiation and effective sun protective measures to protect themselves and young children is currently unknown. Therefore, this study aims to investigate primary PST's current tanning and sun protection behaviours, awareness of the dangers related to UV exposure and perceived knowledge and skill to be able to teach sun safety.

## 2 METHODS

### 2.1 Sample/participants

A convenience sample of second and fourth year undergraduate PST’s (n = 555) enrolled in the Bachelor of Primary Education in 2019 at one Western Australian university were invited to be in the study.

### 2.2 Assessment tool

This study used the online Preservice Teacher Sun Safety Survey (PSTSSS), which was designed for the purposes of this study. The PSTSSS included previously validated questions from state and national surveys.^17^, ^18^, plus additional questions added for the study's PST participant group. The PSTSSS took approximately 15 minutes to complete. The first nine checkbox questions of the PSTSSS were used to collect participants’ demographic information and self‐reported skin sensitivity. Following this, there were 21 questions that investigated participants’ sun behaviours, UV radiation awareness and perceptions of school‐based sun safety. Responses were provided on a 5‐point Likert scale and ranged from “never” to “everyday” for behavioural frequency variables and from “strongly disagree” to “strongly agree” for attitudinal variables. Qualtrics (Utah, UT) software was used to create and administer the PSTSSS via QR code and website links.

### 2.3 Procedures

Approval to conduct this research was granted by the relevant University Human Research Ethics Committee (HREC‐22500). In March 2019, all second and fourth year undergraduate students enrolled in a Bachelor of Primary Education at one Western Australian University were invited to attend an information session that provided a study overview and explained consent processes and what participation would entail. Students also received consent and information letters highlighting that participation in the study was voluntary and unrelated to their curriculum, assessments or degree progression. Consenting PSTs were emailed a QR code and link to the PSTSSS (Qualtrics survey).

### 2.4 Analysis

Survey data were downloaded from Qualtrics and analysed using IBM SPSS Statistics 25 (SPSS Inc Chicago, IL) software. Descriptive analyses assessed frequency and mean data. Chi‐square and logistic regression were used to explore univariate associations between PSTs sun protective behaviours, perceived knowledge and skill to teach sun safety and other demographic independent variables (age, gender, undergraduate year and skin sensitivity) as previous research has shown these to be linked.^19^ Univariate associations with a *p* value of ≤ 0.25 were entered into multivariable logistic regression models using both backward elimination and stepwise variable selection to determine model stability.^20^ Six regression models were developed: Shade usage (no use/use), sunscreen usage (no use/use), hat usage (no use/use), UV index usage (no use/use) and perceived knowledge (do not have/have); perceived skill (do not have/have). For regression analysis, significant factors were combined as completed in previous studies.^19^, ^21^, ^22^ Use of sun protection (ie, shade, sunscreen, hat, UV index), responses were coded as scores of: 1 = no use; ≥2 = use); skin sensitivity: 1‐2 = burn; 3 = do not burn; age: 1 = young adult aged <24 years, 2 = older adult aged ≥25 years; knowledge: <3 = do not have, ≥4 = have; skill: <3 = do not have, ≥4 = have.

## 3 RESULTS

The proportion of PSTs who consented to be involved in the study was 284/555 (51.1%), which was considered average in comparison to previous studies that have investigated university student response rates in the United States^23^ and Australia.^24^, ^25^, ^26^ Nine participants were excluded due to missing data. A final sample of n = 275 (age range = 18‐54; median age = 21 years; second years = 227 (82.5%); fourth years = 46 (16.7%) were included in the analyses (See Table 1). Most participants were female (81.1%), Australian‐born (79.6%) and spoke English at home (97.1%), and attended both primary and secondary school in Australia (88.4%).

**TABLE 1:** Demographics of preservice teacher sample (n = 275)

Survey item	n	%
Student year group		
Second year student	227	82.5
Fourth year student	46	16.7
Missing	2	0.7
Age		
<24 years	207	75.3
25‐29 years	38	13.8
30+ years	30	10.9
Gender		
Males	52	18.9
Females	223	81.1
Country of birth		
Australia	219	79.6
Other	56	20.4
Cultural background		
Australian	187	6
Aboriginal	1	0.4
Asian	6	2.2
European	50	18.2
Middle Eastern	3	1.1
African	13	4.7
Other	14	5.1
Prefer not to say	1	0.4
Language spoken at home most frequently		
English	267	97.1
Other	8	2.9
Schooling in Australia		
Primary only	2	0.7
Secondary only	16	5.8
Both primary and secondary	243	88.4
Neither, I attended outside Australia	14	5.1

### 3.1 Self‐reported sun sensitivity and sun‐related behaviours

Skin sensitivity was assessed by asking about participants’ skin's response if they stayed in the sun unprotected for 30 minutes: 27.6% (n = 76) indicated they would not tan, just burn, 37.8% (n = 104) said they would burn first, then tan and 34.6% (n = 76) said they would not burn, just tan. PSTs that reported their skin would burn in the sun were more likely to seek shade (χ^2^ = 5.90, OR = 0.33, *P* < .02), wear a hat (χ^2^ = 4.02, OR = 0.41, *P = *.05) more frequently than those who reported their skin does not burn (See Table 2).

**TABLE 2:** Multivariable logistic regression of preservice teachers’ sun behaviours and perceptions

Dependent variable	Predictor		*B*	*SE*	χ^a^	OR	95% CI	*p*
Shade^a^	Skin	Burn	−1.1	0.46	5.90	0.33	0.14‐0.81	.02
		Do not burn	1					
Sunscreen^a^	Undergraduate year	Second	−2.23	0.88	7.23	0.93	0.17‐0.65	<.01
		Fourth	1					
Hat^a^	Skin	Burn	−0.9	0.45	4.02	0.41	0.17‐0.98	.05
		Do not burn	1					
Skill^b^	Gender	Males	−0.72	0.32	5.17	0.49	0.26‐0.91	.02
		Females	1					
	Skin	Burn	0.60	0.26	5.02	1.80	0.75‐3.07	.03
		Do not burn	1					

Note

Age, gender, undergraduate year and skin sensitivity were not predictors of UV index use (*P* > .05) or perceived knowledge of sun safety (*P* > .05).

^a^ No use/use.

^b^ Do not have/have.

When asked if they had been sun burnt in the past 12 months, over half of the participants reported they never/rarely got sunburnt (52.3%). However, 39.6% reported they got burnt “sometimes” and 8% stating they got burnt “often/everyday.” A total of 55.6% reported that in the last 12 months they attempted to get a tan; 27.6% reported they “sometimes” tried tanning and 16.8% reported they attempt to get a tan often/everyday (See Table 3). Self‐reported skin sensitivity and tanning frequency findings in this study were reasonably consistent with findings from previous Australian studies.^21^, ^27^

**TABLE 3:** Preservice teachers sun‐related behaviours

Question	Never N (%)	Rarely N (%)	Sometimes N (%)	Often N (%)	Everyday N (%)
In the last 12 months, I got sunburnt	21 (7.6)	123 (44.7)	109 (39.6)	21 (7.6)	1 (0.4)
In the last 12 months, I attempted to get a tan	71 (25.8)	82 (29.8)	76 (27.6)	45 (16.4)	1 (0.4)
I seek shade from 10 AM to 2 PM	22 (8.0)	46 (16.7)	98 (35.6)	91(33.1)	18 (6.5)
I wear sunscreen	6 (2.2)	31 (11.3)	113 (41.1)	104 (37.8)	21 (7.6)
I wear a hat	22 (8.0)	66 (24.0)	102 (37.1)	73 (26.5)	12 (4.4)
I check the UV index	83 (30.2)	91 (33.1)	69 (25.1)	26 (9.5)	5 (1.8)
I use UV index	97 (35.3)	90 (32.7)	64 (23.3)	19 (6.9)	4 (1.5)

Less than 10% of participants reported they used sun protective measures daily (midday shade use: 6.5%; sunscreen: 7.6%; hat: 4.4%). The most common response for all three sun protective measures was that the participants “sometimes” protected themselves from the sun (seeking midday shade: 38.9%; sunscreen: 41.1%; hat: 37.1%). Under 50% of participants reported using each sun protection measure often/everyday. Participants were more likely to use sunscreen (45.4%) or shade from 10 AM to 2 PM (39.6%) often/everyday, compared to wearing a hat often/everyday (30.9%). Second year PSTs were more likely to wear sunscreen than fourth year PSTs (χ^2^ = 7.23, OR 0.93, *P* < .01). Most participants did not use the UV Index to aid sun protection: 63.3% rarely/never checked the UV index and 25.1% did so sometimes, while 68.0% rarely/never use the UV index as a sun protection tool. Age, gender, undergraduate, year and skin sensitivity were not predictors of UV index use (*P* > .05).

### 3.2 Sun protection attitudes

Table 4 shows just under half the PST’s (48.7%) agreed they understood the UV index and 7.6% strongly agreed they understood it, while 29.5% were neutral and 13.8% reported they did not understand it. Most participants agreed/strongly agreed that the UV index is a useful resource to assist with sun protection (73.1%) and that understanding it is important to properly protect themselves (69.1%). Participants almost unanimously agreed/strongly agreed it was important for teachers to thoroughly understand sun safety to enable them to properly educate their students (96.4%). However, more than half of the PSTs (60%) felt they did not have the required skills to teach sun safety to properly protect young children while at school with 38.5% reporting they did not have the required knowledge. Regression analyses revealed that gender (χ^2^ = 5.17, OR = 0.49, *P* = .02) and skin sensitivity (χ^2^ = 5.02, OR = 1.80, *P* = .03) were the strongest predictors of perceived skill to be able to teach sun safety. However, age, gender, undergraduate year and skin sensitivity were not predictors of perceived knowledge of sun safety (*P* > .05).

**TABLE 4:** Preservice teachers’ perceptions of UV and sun protection

UV attitudes	Strongly disagree N (%)	Disagree N (%)	Neutral N (%)	Agree N (%)	Strongly agree N (%)
I understand the UV index	2 (0.7)	36 (13.1)	81 (29.5)	134 (48.7)	21 (7.6)
I feel that understanding UV index is important	5 (1.8)	7 (2.5)	72 (26.2)	146 (53.1)	44 (16.0)
I feel the UV index is useful	3 (1.1)	7 (2.5)	63 (22.9)	162 (58.9)	39 (14.2)
Teachers are responsible for ensuring students are UV protected while at school	1 (0.4)	3 (1.1)	50 (18.2)	149 (54.2)	68 (24.7)
It is important that primary teachers understand sun safety well so they can properly educate their students	2 (0.7)	0 (0.0)	4 (1.5)	116 (42.2)	149 (54.2)
I feel I have the knowledge to teach sun safety in primary schools	2 (0.7)	36 (13.1)	127 (46.2)	87 (31.6)	19 (6.9)
I feel I have the skills to teach sun safety in primary schools	2 (0.7)	35 (12.7)	124 (45.1)	96 (34.9)	14 (5.1)

## 4 DISCUSSION

National evaluation research has supported a strong economic case for skin cancer prevention in Australia.^28^ Funding for skin cancer prevention varies considerably across the country. While treatment for skin cancer in Western Australia is estimated to cost $90 million per annum^29^, the state government funding for skin cancer prevention averaged just $734,000 per annum over the past 3 years. Research from three national surveys between 2003 and 2011 suggest that there has been a reduction in pro‐tanning attitudes and behaviours among Australians.^30^ However, recent findings in WA indicate a slight increase in tanning and sun burn rates among adolescents and adults in recent years,^31^ which potentially highlight a need for increased state‐wide prevention and targeted education campaigns to curb this rise.

Research indicates that since Cancer Council Australia launched the national SunSmart Schools Program (SSSP) in 1998, there was significant uptake by primary schools across the country, growing from 19% in 1998 to an estimated 71% in 2018; and also concluded that that schools registered in the SSSP had greater likelihood of implementing policies and practice.^32^ However, current registration in the SSSP^3^ and implementation of policy is less than desirable in WA. Cancer Council WA data (as of July 2020), indicates that only 36% of WA Primary Schools are registered in the SSSP. Furthermore, research in 2017 showed that 32% of WA primary schools did not have a written sun protection policy.^33^ While online resources and professional learning materials are available to all teachers via the Generation SunSmart website, there is a clear need increase SSSP uptake and policy implementation in WA to improve schools sun protection approaches. Additionally, it remains unknown what level of understanding primary teachers have of UV radiation and effective sun protective measures for primary‐aged children and how policies are being implemented and adhered too.

To our knowledge, the present study is the first to assess sun safety behaviours, knowledge and attitudes among primary PSTs. While age and gender were not predictors of sun protective behaviours (shade, sunscreen, hat or UV index usage), participants who reported that their skin would burn in the sun were more likely to seek shade (*P* = .02) and wear hats (*P* = .05) more frequently than those who reported their skin does not burn. These findings are consistent with previous studies that have shown individuals with more sensitive skin are commonly more vigilant with sun protective measure.^21^, ^34^

While few PSTs actively tanned or sunburned on a regular basis, many did not use sun protection regularly. Similar results have been found for the broader WA community with 18‐45 year old's reporting only moderate levels of use of sun protection with sunscreen the most common method of protection^19^. Given the participants’ infrequent use of sun protection in the present study, school policies encouraging and reinforcing sun protection for staff, as well as students, are likely to be important to support positive behaviour change when this cohort start teaching.^7^

Our findings indicate that primary PST’s saw the UV Index as an important and useful tool to protect themselves from harmful UV radiation, however, few used it regularly and over 40% reported they did not properly understand it or were unsure. Similarly, other studies have shown that Australian populations were reasonably aware of the UV Index, but that it was not widely used or correctly understood.^6^, ^16^, ^35^, ^36^ Evaluations of public education campaigns in adults indicate they can successfully increase awareness and understanding of the UV Index by the general public^18^, so it is likely that targeted approaches with primary PST’s could also be effective.

Primary teachers play an important and influential role as mentors and role models for many young children ^12^, ^13^, ^37^ at a time when sun exposure can have a significant impact on the development of future cancer risk.^38^ Over 18% of participants provided a “neutral” response to believing it was their responsibility to ensure students are UV protected while at school, potentially indicating they were unsure. This is concerning as primary teachers are responsible for children during the part of the day when UV radiation is at its peak.^39^ Our findings highlight that UV radiation and sun protection knowledge among primary PSTs is lower than ideal and potentially highlight a need to enrich existing initial teacher education programs to include more sun safety content.

Participants in this study had varying levels of confidence about their abilities to teach Sun Safety effectively, in spite of their acknowledgement of the importance of sun protection in schools. This has implications for the effectiveness and consistency of application of sun protection practices and teaching in Australian schools. Previous research indicates that school teachers are in a prime position to influence child health and for Health Education to be effective in primary schools, teachers must receive specific training.^12^, ^40^

Studies indicate that exiting PSTs perceive they are more knowledgeable and skilled than PSTs entering their degrees.^41^, ^42^ However, our findings indicated that other than sunscreen usage (*P* < .01), undergraduate year was not a predictor for other sun protective behaviours, perceived knowledge or perceived skill to be able to teach sun safety. In contrast, our findings indicate that many PST’s in both second and fourth year feel that they do not have the perceived knowledge or skill to effectively teach sun safety to their future students there is a need for targeted interventions specially designed for PST’s to raise awareness and upskill PST’s on effective UV radiation protective measures.

This study was novel as for the first time it investigated primary PST’s tanning, sun protective behaviours as well as their perceptions of UV radiation and confidence and competence to be able to teach sun safety. However, there were several limitations to this research. First, due to feasibility restrictions, a convenience sample of only second and fourth year PST’s from one Western Australian university were included in this study. Second, the response rate was 51.1% with only 284 of the invited 555 PSTs providing informed consent to be involved in the study. It is unknown if PSTs who chose not to be involved in the study would have responded differently to certain questions in the survey. Third, the sample was relatively homogeneous with regards to age and gender, and hence, the findings may not be generalisable across all PSTs at other Australian Universities. Finally, a self‐report measure was used to collect data, and hence, there is the potential for reporting bias.

## 5 CONCLUSION

Survey data indicated that there are inconsistent understandings of UV radiation, tanning and sun protective behaviours among primary PST’s. Many PST’s felt they lacked the required knowledge or skills to properly teach children about the dangers of UV radiation and sun safety. Our findings highlight a need for greater UV radiation and sun safety education at tertiary level focusing on effective sun protective behaviours for primary‐aged children. Within existing teacher education programs, a targeted intervention specially designed for primary PSTs is warranted.

## CONFLICT OF INTEREST

There are no competing interests.

## AUTHORS CONTRIBUTIONS

All authors are responsible for reported research and have participated in the concept and design, data collection, analysis and interpretation of data, drafting or revising, and have approved this manuscript as submitted.

## ENDNOTES

^1^
Refers to PSTs that are studying a Bachelor of Secondary Education.

^2^
Refers to PSTs that are studying a Bachelor of Primary Education.

^3^
Re‐registration of SSSP is required every 3 years in WA.
